# Differential patterns of lifestyle behaviors among low- and high-income postmenopausal women in Korea: a latent class analysis

**DOI:** 10.1186/s12905-023-02767-5

**Published:** 2023-11-18

**Authors:** Haein Lee, Bo Gyeong Lee, In Seo La

**Affiliations:** 1 College of Nursing, Daegu Catholic University, 33 Duryugongwon-ro 17-gil, Nam-gu, Daegu, 42472 Republic of Korea; 2 College of Nursing, Daegu Catholic University, 33 Duryugongwon-ro 17-gil, Nam-gu, Daegu, 42472 Republic of Korea; 3 College of Nursing Science, Kyung Hee University, 26, Kyungheedae-ro, Dongdaemun-gu, Seoul, 02447 Republic of Korea

**Keywords:** Postmenopause, Healthy lifestyle, Latent class analysis, Income, Socioeconomic disparities in health

## Abstract

**Background:**

Healthy lifestyle behaviors among postmenopausal women are important to prevent chronic diseases and improve health later in life. Heterogeneous lifestyle patterns may exist among postmenopausal women, and socioeconomic status (SES) is a critical determinant of lifestyle behaviors. However, little is known about distinct SES-specific patterns of lifestyle behaviors among postmenopausal women. Thus, this study used latent class analysis to identify subgroups of postmenopausal women with different health behaviors according to income and to examine the predictors of income-specific subgroups.

**Methods:**

We analyzed nationally representative data from the Eighth Korea National Health and Nutrition Examination Survey, collected in 2019 and 2020. We used nine lifestyles (i.e., current smoking and drinking, high-risk drinking, walking, muscle-strengthening exercise, sleep, vegetable and fruit intakes, and weight control efforts). We conducted a multiple-group latent class analysis using monthly household income as a proxy for SES. The monthly household income variable was calculated by standardizing monthly household income by the number of family members and then divided into quintiles. We classified the participants into low- (i.e., Q1 and Q2) and high-income (i.e., Q3, Q4, and Q5) groups.

**Results:**

Although the three-class models best fit the data of low- and high-income groups, we found differential patterns by income: (a) for low-income group, “relatively healthy (RH),” “lowest physical activity, insufficient fruit intake, and no intention to control weight,” and “high-risk drinking and insufficient fruit intake” classes and (b) for high-income group, “RH,” “lowest physical activity,” “high-risk drinking and insufficient fruit intake and sleep” classes. The proportion of the RH class was largest in both groups. However, lifestyle patterns in low-income group showed multiple and unhealthy characteristics than those in high-income group.

**Conclusions:**

This study suggests that different underlying lifestyle patterns exist in postmenopausal women with low- and high-income. To promote healthy behaviors among postmenopausal women, health professionals should develop and apply lifestyle interventions tailored to lifestyle pattern characteristics according to income.

## Introduction

As life expectancy increases, most women spend more than a third of their lives in menopause [1]. By 2025, there will be 1.1 billion postmenopausal women worldwide [2]. In Korea, the number of postmenopausal women is expected to increase to 6 in 10 by 2060 [3]. Although menopause is a developmental transition, postmenopausal women experience physical, psychological, and social changes due to estrogen and androgen reduction or imbalance related to the loss of ovarian follicular activity, with both short- and long-term consequences [1, 4]. Specifically, they may experience (a) hot flashes, night sweats, mood changes, genitourinary syndrome (e.g., urinary incontinence, vaginal dryness, and dyspareunia), and sleep disorders in the short term, and (b) low quality of life or chronic diseases such as diabetes, cardiovascular disease, and osteoporosis in the long term [5–8]. Women’s health during this period determines successful and healthy aging [9].

Current literature indicates that healthy lifestyle behaviors in postmenopausal women are a critical window for preventing chronic diseases, enhancing their quality of life, and improving their health and function in later life [6, 10, 11]. Previous reviews have found that healthy eating habits improve cardiovascular and metabolic health and reduce the incidence of breast cancer [12, 13]. In addition, low physical activity, smoking, and obesity have been shown to increase the overall cardiovascular disease incidence and all-cause mortality [14]. Therefore, a sufficient understanding of lifestyle behaviors among postmenopausal women is necessary [6].

According to the health lifestyle theory [15], health lifestyle refers to collective patterns of health behaviors that people have adopted based on their life opportunities or circumstances. Although individuals choose their lifestyles, the individual choices are determined by the norms, values, and social environments shared by a particular group or social class [15–17]. This theory presents class circumstances (i.e., socioeconomic status [SES]) as the most important structural factor influencing lifestyle formation [15, 16]. Empirical studies have consistently indicated a significant relationship between lifestyle and SES in postmenopausal women. For example, a study conducted in the U.S. [18] found that higher household income was associated with higher physical activity. Additionally, a nationally representative study in Korea found that lower household income was associated with high-risk alcohol consumption [19]. Given that heterogeneous lifestyle patterns exist and SES is an important determinant of these patterns [15, 20], it is critical to identify lifestyle patterns according to SES among postmenopausal women.

To identify lifestyle patterns, recent researchers have used latent class analysis (LCA) across different age groups. For example, two systematic reviews identified the predominant patterns of obesity-related risky lifestyles in children and adolescents [21] and substance use behaviors in young adults [22], respectively. In addition, Zhang [23] identified health lifestyle patterns among Chinese older adults [23]. LCA, a person-centered approach, is useful for classifying individuals who share unobserved latent characteristics [24]. Latent class models (LCMs) assume a mutually exclusive underlying set of subgroups (i.e., latent classes) based on individual response patterns to their categorized behavioral characteristics [24]. LCMs enable an increase in the effectiveness of interventions by offering tailored intervention programs based on multidimensional individual characteristics [25]. However, previous studies on postmenopausal women’s lifestyles have primarily focused on (a) one particular lifestyle and (b) its’ linear relationships with risk factors based on variable-centered approaches [18, 19, 26–28].

Moreover, a thorough understanding of the influence of SES on postmenopausal women’s lifestyles is essential for understanding the disparities in their lifestyles and subsequent health outcomes [29]. Therefore, in addition to identifying LCMs of lifestyle in postmenopausal women, it is important to identify SES-specific LCMs. However, LCA studies of postmenopausal women’s lifestyles have assumed the same LCMs across SES levels [10, 30–32]. Although the previous LCA studies of postmenopausal women identified two to five homogenous latent classes (e.g., healthier, poor diet, low physical activity, substance use, and risky-lifestyle classes) [10, 30–32], there are few studies that examine whether the LCMs among low- and high-SES groups are comparable. This makes it difficult to provide detailed information on latent class differences between low- and high-SES groups [24].

This study aimed to investigate lifestyle patterns using LCA and the predictors of lifestyle patterns among low- and high-SES postmenopausal women. Given that household income affects lifestyle, health outcomes, and quality of life of postmenopausal women [18, 19, 33–35], we used household income as an indicator of SES [15]. Therefore, based on the health lifestyle theory and the aforementioned studies indicating the close relationship between income and lifestyles [15, 17–19], we hypothesized that (i) distinctive lifestyle patterns would be identified between the low- and high-income postmenopausal women and (ii) the predictors of lifestyle patterns would be different across the two groups.

## Methods

### Data and participants

We used secondary data from the Eighth Korea National Health and Nutrition Examination Survey (KNHANES VII) collected in 2019–2020. The KNHANES is a nationally representative and cross-sectional data collected by the Korea Disease Control and Prevention Agency (KDCA) since 1998. The purpose of the KNHANES is to identify the population-based health status, health behaviors, and nutritional intake [36]. To recruit a nationally representative sample, the KDCA selected participants using stratified multistage cluster sampling. Specifically, KNHANES VII (a) divided 192 primary sampling units (PSUs) per year based on administrative district, residential place (i.e., rural or urban), and housing type, and (b) selected 25 households from each PSU.

**Fig. 1:**
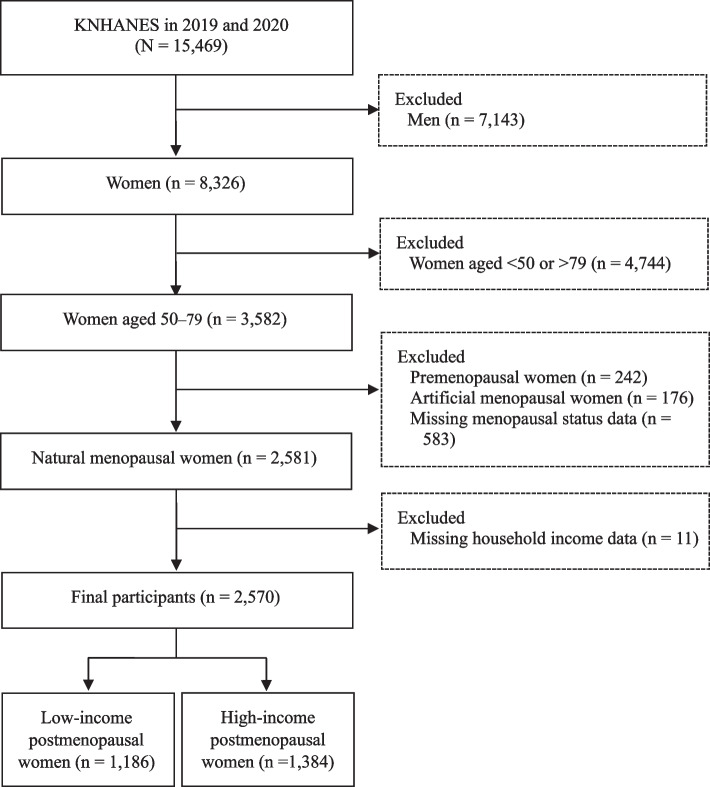
Flow Chart of the Study Participants

The data from the KNHANES conducted included 15,469 participants. Of the 3582 women aged 50–79 years, 2581 who reported natural menopause were selected. Premenopausal and artificially menopausal women were excluded. Our data were limited to postmenopausal women who reported their household income. The health lifestyle theory states that upper and upper-middle class lifestyles are generally healthier [15]. Therefore, we used the household income variable which was monthly household income standardized according to the number of family members (i.e., monthly household income divided by the square root of the number of household members) and classified into quintiles, from the lowest (Q1) to the highest (Q5) based on the raw data. We dichotomized the variable into low-income (i.e., Q1 and Q2) and high-income (i.e., Q3, Q4, and Q5). Finally, we included 2570 postmenopausal women, including 1186 low-income and 1384 high-income women (Fig. 1).

## Measures

### Indicators of lifestyle behaviors

We selected nine lifestyle factors associated with health-related quality of life and chronic diseases in postmenopausal women: current smoking, current and high-risk drinking, insufficient walking and muscle-strengthening exercises, insufficient sleep duration, nondaily vegetable and fruit intakes, and lack of weight control efforts [13, 37–40]. Considering that identification problems may exist with more parameters to be estimated in LCA [24], we dichotomized all indicators.

Substance use included tobacco product and alcohol use. Current smoking status was measured using six questions about the participants’ lifetime and current tobacco product use (i.e., combustible cigarette [CC], electronic cigarette [EC], and heated tobacco product [HTP] use). Current CC use was defined as having smoked 100 or more CCs in their lifetime and currently smoking CCs [41], current EC use was defined as having used ECs in their lifetime and in the past 30 days, and current HTP use was defined as having used HTPs in their lifetime and currently using HTPs. Based on the information on current smoking status, the current use of at least one tobacco product among CC, EC, and HTP was defined as current smoking. Current drinking was assessed by asking the participants if they had consumed alcohol at least once a month in the past year. High-risk drinking was defined as the consumption of five or more alcoholic drinks on the same occasion at least once a month [42].

For physical activity, two dichotomous items were included: walking and muscle-strengthening exercises. We defined insufficient walking as not walking for more than 30 min at least five days per week, and insufficient muscle-strengthening exercise as not performing muscle-strengthening exercises (e.g., lifting weights, sit-ups, and push-ups) two or more days per week [43]. In addition, two continuous variables were used for sleep duration: the average self-reported hours of sleep on weekdays and weekends. We estimated average sleep duration using the following formula: 5/7 × average weekday sleep duration + 2/7 × average weekend sleep duration. Insufficient sleep duration was defined as less than seven hours of sleep per day on average [44]. For dietary behavior, we included vegetable and fruit consumption. Consumption of vegetables, including kimchi (i.e., a traditional Korean fermented vegetable food), and fruits less than once a day in the past year were classified as nondaily vegetable and fruit intakes, respectively. Finally, weight control efforts were assessed using the following question: “Have you tried to control your weight intentionally in the past year?” A lack of weight control effort was defined as no intentional effort to control weight in the past year.

### Predictors of latent class membership

Based on the literature on postmenopausal women’s lifestyle [10, 18, 30–32], we used four demographic variables and two chronic diseases as predictors. First, demographics included age (< 65 and ≥ 65 years), education (high school graduation or lower and two-year college or higher), living with a spouse (yes and no), and employment status (unemployed and employed), which were measured using single items.

Second, hypertension and diabetes were included as representative chronic diseases in postmenopausal women. Hypertension was defined as (a) a systolic blood pressure level of 140 mmHg or higher, (b) a diastolic blood pressure level of 90 mmHg or higher, or (c) self-reported use of antihypertensive medication [45]. Diabetes was defined as (a) a fasting glucose level of 126 mg/dL or greater, or (b) self-reported use of diabetes medication [46].

### Data analysis

We analyzed secondary data using SAS Version 9.4. First, to identify the sample characteristics by household income, we used descriptive statistics incorporating a complex sampling design (i.e., strata, weight, and primary sampling units) and domain analysis [47]. Second, to estimate the latent lifestyle classes among low- and high-income groups, we used PROC LCA Version 1.3.2 [48]. We tested the LCMs with one to five latent classes for the entire sample. To select the best-fitting model, we compared the likelihood-ratio statistics (*G*^2^), Akaike information criterion (AIC), Bayesian information criterion (BIC), adjusted BIC, entropy, and log-likelihood values. Entropy is defined as the degrees of uncertainty, and higher values indicate better latent class separation [24]. Higher values of entropy and lower values of *G*^2^, AIC, BIC, adjusted BIC, and log-likelihood indicate a better model fit [24]. Additionally, we considered the percentage of seeds associated with the best-fitting model, interpretability, and parsimony of each model. The higher percentage of seeds associated with the best-fitting model presents the better identified model [24]. Once the best-fitting model was determined for the entire sample, we evaluated the measurement invariance across low- and high-income groups. We estimated (a) a model with free estimation of item response probability parameters and (b) a model with constrained same item response probability parameters across groups. We then conducted a *G*^2^ difference test and determined measurement invariance to be established when the results did not show a significant difference [48]. A significant difference implied that the measurement invariance was violated, requiring separate estimation of the LCMs across groups [49]. Third, we identified associations between predictors and latent class membership using a multivariate analysis. We conducted a multinomial logistic regression analysis to investigate the relationships between the predictors and class membership among low- and high-income groups [49]. Four demographics and the health status of participants (i.e., with or without hypertension and/or diabetes) were included as predictors.

## Results

### Sample characteristics

For the low-income group, 59.6% were aged 65 years or older, and 6.9% had a two-year college or higher education. Approximately 61% resided with their spouse and 39.9% were employed. The prevalence of hypertension and diabetes among low-income participants were 54.2 and 21.3%, respectively. In the high-income group, 23.0% were aged 65 years or older, and 23.8% had a two-year college or higher education. Approximately 82% resided with their spouse, and 50.3% were employed. The prevalence of hypertension and diabetes among high-income participants were 38.3 and 14.4%, respectively. Detailed information on the sample characteristics is provided in Table 1.

**Table 1:** Characteristics of Low- and High-income Postmenopausal Women (*N* = 2570)

Factor	Frequency (%)^a^			
	Low-income (*n* = 1186)		High-income (*n* = 1384)	
Age				
< 65	396	(40.4)	1000	(77.0)
≥65	790	(59.6)	384	(23.0)
Education				
High school graduation or lower	1112	(93.1)	1060	(76.2)
Two-year college or higher	72	(6.9)	323	(23.8)
Living with a spouse^b^				
Yes	689	(60.7)	1116	(82.4)
No	496	(39.3)	267	(17.7)
Employment status				
Employed	470	(39.9)	696	(50.3)
Unemployed	715	(60.1)	687	(49.8)
Hypertension				
Yes	671	(54.2)	553	(38.3)
No	512	(45.8)	824	(61.7)
Diabetes				
Yes	266	(21.3)	202	(14.4)
No	892	(78.7)	1161	(85.6)
Current smoking^b^				
Yes	56	(5.1)	33	(2.2)
No	1128	(94.9)	1349	(97.8)
Current drinking^b^				
Yes	263	(22.9)	439	(33.1)
No	921	(77.2)	943	(66.9)
High-risk drinking^b^				
Yes	91	(9.1)	135	(9.9)
No	1093	(90.9)	1247	(90.1)
Insufficient walking				
Yes	745	(62.4)	758	(54.9)
No	438	(37.6)	625	(45.1)
Insufficient muscle-strengthening exercises				
Yes	1059	(88.8)	1169	(84.1)
No	127	(11.2)	214	(15.9)
Insufficient sleep duration^b^				
Yes	637	(53.5)	681	(47.9)
No	545	(46.5)	703	(52.2)
Nondaily vegetable intake^b^				
Yes	3	(0.3)	5	(0.4)
No	1040	(99.8)	1167	(99.6)
Nondaily fruit intake				
Yes	541	(52.3)	431	(36.9)
No	502	(47.7)	741	(63.1)
Lack of weight control efforts				
Yes	473	(38.8)	376	(25.5)
No	711	(61.2)	1006	(74.5)

^a^Unweighted frequency and weighted percentage

^b^Percentages may not total 100.0 due to rounding

### Patterns of lifestyle behaviors among low- and high-income groups

Before estimating the LCMs across household income-specific groups, we estimated the LCMs of lifestyle behaviors using the entire sample. As the number of latent classes increased, the values of *G*^*2*^ and AIC decreased. However, the adjusted BIC was the lowest in the three-class model and the level of model identification was poor (i.e., low percentages of models were associated with the best-fit model) in the four- and five-class models. Considering the parsimony and easy interpretability of the models, we selected the three-class model. To test measurement invariance across low- and high-income groups, the model with measurement invariance imposed (*G*^*2*^ = 430.88, *df* = 992) was compared to the model without measurement invariance imposed (*G*^*2*^ = 375.97, *df* = 965). The results showed that measurement invariance across household income was violated (Δ*G*^*2*^ = 54.91, *df* = 27, *p* = .001). Thus, we estimated the LCMs separately for each income group [49].

We compared the fit statistics of one- to five-class models among low- and high-income groups. In both groups, the values of *G*^*2*^ and AIC decreased as the number of latent classes increased. However, the adjusted BIC indicated that the three-class model fit better than the four- and five-class model, and the differences in *G*^*2*^ and AIC among three-, four-, and five-class models were not substantial. Furthermore, considering the poor level of model identification, interpretability, and parsimony in the four- and five-class models, we selected the three-class models for both groups (Table 2).

**Table 2:** Fit Statistics of Latent Classes of Lifestyle Behaviors Among Postmenopausal Women

Number of latent classes	*G*^*2*^	Degree of freedom	AIC	BIC	Adjusted BIC	Entropy	Log-likelihood	Percentage of seeds associated with best fitted model
Total (n = 2570)								
1	1091.61	502	1109.61	1162.27	1133.68	1.00	−10,381.94	100.0%
2	398.84	492	436.84	548.02	487.65	0.93	−10,035.56	100.0%
**3**	**250.01**	**482**	**308.01**	**477.71**	**385.57**	**0.54**	**− 9961.14**	**98.0%**
4	222.26	472	300.26	528.47	404.56	0.60	− 9947.26	18.0%
5	200.54	462	298.54	585.27	429.59	0.60	− 9936.41	34.0%
Low-income (n = 1186)								
1	579.12	502	597.12	642.82	614.23	1.00	− 4711.93	100.0%
2	245.99	492	283.99	380.48	320.13	0.95	− 4545.37	100.0%
**3**	**187.43**	**482**	**245.43**	**392.71**	**300.59**	**0.54**	**− 4516.09**	**96.0%**
4	165.49	472	243.49	441.55	317.67	0.62	− 4505.12	26.0%
5	145.29	462	243.29	492.13	336.49	0.66	− 4495.02	6.0%
High-income (n = 1384)								
1	644.65	502	662.65	709.75	681.16	1.00	− 5581.17	100.0%
2	255.39	492	293.39	392.81	332.46	0.86	− 5386.54	100.0%
**3**	**188.52**	**482**	**246.52**	**398.27**	**306.15**	**0.52**	**− 5353.10**	**94.0%**
4	170.29	472	248.29	452.37	328.48	0.60	− 5343.99	14.0%
5	155.41	462	253.41	509.82	354.16	0.54	− 5336.55	14.0%

Bold letters indicate the best fitting models. Higher values of entropy and lower values of G2, AIC, BIC, adjusted BIC, and log-likelihood indicate a better model fit. The higher percentage of seeds associated with the best-fitting model presents the better identified model

*G*^2^ the likelihood-ratio statistic, *AIC* Akaike’s information criterion, *BIC* Bayesian information criterion

For the low-income group, we labeled the classes as “relatively healthy (RH; 45.8%),” “lowest physical activity, insufficient fruit intake, and no intention to control weight (LP-IFW; 45.5%),” and “high-risk drinking and insufficient fruit intake (HD-IF; 8.8%).” Those in the RH class had lower probabilities for most indicators than those in the other classes. Participants in this class had the lowest probabilities of insufficient walking (50.1%) and muscle-strengthening exercises (83.2%). The LP-IFW class was characterized by the highest probabilities of insufficient walking (76.5%) and muscle-strengthening exercises (96.6%), and those in the LP-IFW class had the highest probabilities of nondaily fruit intake (70.7%) and weight control efforts (55.3%). The HD-IF class comprised participants with higher probabilities of current (97.6%) and high-risk drinking (87.2%) than the other classes.

Three classes for the high-income group were labeled as “RH (46.0%),” “lowest physical activity (LP; 40.7%),” “high-risk drinking and insufficient fruit intake and sleep (HD-IFS; 13.3%).” In the RH class, participants had less than 50% probabilities for all indicators except insufficient muscle-strengthening exercises (73.6%). Those in the LP class had the highest probability of insufficient walking (76.3%) and muscle-strengthening exercises (96.9%). The HD-IFS class was characterized by the highest probabilities of current drinking (98.4%) and high-risk drinking (73.3%); those in this class had higher probabilities of insufficient sleep (59.5%) and fruit intake (65.6%) than the other classes (Table 3).

**Table 3:** Item-Response Probabilities of Lifestyle Behaviors Among Low- and High-income Postmenopausal Women (*N* = 2570)

	Low-income (n = 1186)			High-income (*n* = 1384)		
	RH	LP-IFW	HD-IF	RH	LP	HD-IFS
Probability of membership	0.458	0.455	0.088	0.460	0.407	0.133
Current smoking	0.007	0.053	0.227	0.000	0.032	0.081
Current drinking	0.189	0.110	**0.976**	0.215	0.216	**0.984**
High-risk drinking	0.000	0.000	**0.872**	0.000	0.000	**0.733**
Insufficient walking	**0.501**	**0.765**	**0.604**	0.342	**0.763**	**0.603**
Insufficient muscle-strengthening exercises	**0.832**	**0.966**	**0.834**	**0.736**	**0.969**	**0.845**
Insufficient sleep duration	**0.558**	**0.539**	0.443	0.478	0.474	**0.595**
Nondaily vegetable intake	0.000	0.006	0.000	0.005	0.000	0.015
Nondaily fruit intake	0.305	**0.707**	**0.647**	0.206	0.462	**0.656**
Lack of weight control efforts	0.261	**0.553**	0.328	0.188	0.372	0.255

Bold figures indicate that the item-response probability is 0.50 or above

*RH* Relatively healthy, *LP-IFW* Lowest physical activity, insufficient fruit intake, and no intention to control weight, *HD-IF* High-risk drinking and insufficient fruit intake, *LP* Lowest physical activity, *HD-IFS* High-risk drinking and insufficient fruit intake and sleep

### Predictors associated with class membership

Table 4 presents the associations between predictors and latent class memberships using the RH class as a reference class. In the low-income group, participants aged 65 years or older had a higher risk of belonging to the LP-IFW class (odds ratio [OR] = 5.93) and a lower risk of belonging to the HD-IF class (OR = 0.38). Those with a two-year college education or higher were less likely to belong to the LP-IFW class (OR = 0.08). Living with a spouse and being unemployed decreased the likelihood of belonging to the LP-IFW and HD-IF classes. Diabetes was associated with an increased likelihood of belonging to the LP-IFW class (OR = 2.09). In the high-income group, those aged 65 years or older had a lower risk of belonging to the HD-IFS class (OR = 0.26). A two-year college or higher education, living with a spouse, and unemployment decreased the likelihoods of belonging to the LP and HD-IFS classes. Hypertension was associated with an increased likelihoods of belonging to the LP (OR = 1.74) and HD-IFS classes (OR = 1.79).

**Table 4:** Predictors of Membership in Latent Class of Lifestyle Behaviors Among Low- and High-income Postmenopausal Women (*N* = 2570)

Predictor	Low-income (n = 1186)					High-income (n = 1384)				
	*p*-value	LP-IFW		HD-IF		*p*-value	LP		HD-IFS	
		OR	95%CI	OR	95%CI		OR	95%CI	OR	95%CI
Age (ref. = < 65)										
≥65	<.001	5.93	(2.36, 14.91)	0.38	(0.23, 0.64)	<.001	0.96	(0.55, 1.68)	0.26	(0.14, 0.47)
Education (ref. = High school graduation or lower)										
Two-year college or higher	.032	0.08	(0.01, 0.55)	0.66	(0.31, 1.37)	<.001	0.40	(0.23, 0.70)	0.32	(0.19, 0.54)
Living with a spouse (ref. = No)										
Yes	<.001	0.40	(0.22, 0.70)	0.38	(0.24, 0.61)	.014	0.54	(0.31, 0.93)	0.46	(0.28, 0.78)
Employment status (ref. = Unemployed)										
Employed	.200	1.53	(0.89, 2.65)	1.57	(0.99, 2.49)	<.001	2.23	(1.39, 3.58)	3.15	(2.03, 4.88)
Hypertension (ref. = No)										
Yes	.202	1.67	(0.97, 2.89)	1.49	(0.94, 2.38)	.022	1.74	(1.09, 2.77)	1.79	(1.16, 2.78)
Diabetes (ref. = No)										
Yes	.008	2.09	(1.17, 3.72)	0.75	(0.40, 1.40)	.138	1.62	(0.92, 2.86)	0.93	(0.51, 1.70)

Reference group = Relatively healthy

*LP-IFW* Lowest physical activity, insufficient fruit intake, and no intention to control weight, *HD-IF* High-risk drinking and insufficient fruit intake, *LP* Lowest physical activity, *HD-IFS* High-risk drinking and insufficient fruit intake and sleep, *OR* Odds ratio, *CI* Confidence interval

## Discussion

This study offers new insights into the lifestyle behavioral patterns of postmenopausal women according to income level as an indicator of SES. The use of LCA provides a better understanding of the role of income in lifestyle patterns than variable-centered approaches. As hypothesized, the classification of the three subgroups differed by income: (a) RH, LP-IFW, and HD-IF classes for low-income group and (b) RH, LP, and HD-IFS classes for their high-income counterparts. Previous LCA studies of postmenopausal women investigating patterns of lifestyle behaviors did not consider differences in patterns by SES [10, 30–32], making it difficult to directly compare the results of previous studies with ours. However, the characteristics of the LCMs in our study were consistent with those reported in previous studies. For example, the proportion of the RH class was the largest in both groups in our study, which was consistent with previous studies on postmenopausal women in the U.S. and Australia [10, 30]. In addition, latent classes in both groups were less likely to engage in sufficient physical activity, especially muscle-strengthening exercises. Similarly, a previous study examining race-specific lifestyle patterns in postmenopausal women found that participants in an Asian group were physically inactive across all classes [10].

Consistent with the health lifestyle theory [15–17], we found differential patterns between low- and high-income groups, and the low-income latent classes showed multiple unhealthy characteristics. When comparing RH and LP-IFW in the low-income group and RH and LP in the high group, to which about 90% of the participants belong in each group, latent classes in the low-income group had higher risks for various unhealthy lifestyles. For example, the RH class in low-income group showed high probabilities of insufficient muscle-strengthening exercises, walking practice, and sleep duration. Unlike the LP class in the high-income group, the LP-IFW class in the low-income group was more likely to have insufficient sleep duration, nondaily fruit intake, and lack of weight control efforts as well as insufficient physical activity. These findings are supported by previous studies indicating that low-income adults are more likely to engage in a greater number of risky lifestyle behaviors than their high-income counterparts [50, 51]. The consistency of our findings with the theoretical and empirical evidence highlights the need to understand lifestyle patterns among postmenopausal women according to their income level.

In terms of predictors, higher education served as a protective factor that reduced the likelihood of unhealthy lifestyle patterns in both groups. These findings are consistent with those of previous LCA studies in postmenopausal and middle-aged women [10, 30, 52]. According to the health lifestyle theory [15–17], these associations between SES and unhealthy lifestyle behaviors may be because those with low income and education levels do not have enough structural context for healthy lifestyle choices or changes. Specifically, those with low SES may (a) lack the ability to access and understand information about healthy lifestyles [20, 53] and (b) have insufficient resources for healthy lifestyles [16, 20, 53]. Moreover, *Pampel* et al. [20] assert that those with low SES are less motivated to engage in healthy behaviors and may engage in unhealthy lifestyle behaviors to cope with higher stress levels.

Latent class memberships in both groups differed by age. Similar to previous studies [54, 55], participants aged 65 years or older had an increased likelihood of belonging to the low-income LP-IFW class but not to the high-income LP class. These findings suggest that the negative impact of age on lifestyle behaviors may be greater in postmenopausal women with low-income. Consistent with the health lifestyle theory [15, 17], the high probability of an unhealthy lifestyle among low-income elderly women may be attributed to the fact that poverty can constrain their healthy lifestyle choices and chances. However, we found that participants aged 65 years or older were less likely to belong to high-risk drinking classes in both groups. These results are consistent with those of previous studies [19, 56, 57]. The two potential reasons for these negative associations between age and high-risk drinking classes across both groups are (a) reduced alcohol consumption due to poor physical health [58] and (b) reduced opportunities for social participation in which alcohol is provided in older age [57].

Living with their spouses was associated with a lower risk of being in the unhealthy classes than the RH class among both groups, which is consistent with previous LCA studies of postmenopausal and midlife women [30, 52]. These results provide evidence that the spouse’s role is important for postmenopausal women to maintain a healthy lifestyle. Given that spousal support is an important health issue for postmenopausal women, previous studies have argued that including a spouse is a critical strategy for maintaining a healthy lifestyle [59, 60].

Regarding the association between LCMs and employment status, employed individuals were less likely to belong to the RH class in both groups. Similarly, a previous LCA study of middle-aged U.S. women found that the proportion of employed women was significantly higher in the drinking class than in the healthy class [52]. The association between employment and unhealthy lifestyle patterns may be partially because employed women have less time to practice a healthy lifestyle [61] and are more exposed to drinking opportunities than their unemployed counterparts [62].

In our study, we found that those with hypertension were more likely to belong to the LP and HD-IFS classes in high-income group, and those with diabetes were more likely to belong to the LP-IFW class in low-income group. These findings may be due to the multiple unhealthy lifestyles in the classes. For example, the LP-IFW class in low-income group had multiple risk factors for diabetes, such as lower physical activity levels, nondaily fruit consumption, and lack of weight control efforts. These associations of lifestyle patterns with hypertension and diabetes are supported by two previous LCA studies of postmenopausal U.S. women: multiple lifestyle and psychosocial risk patterns (a) had the highest proportion of hypertensive patients who had ever been diagnosed with hypertension [10], and (b) had the highest risk of diabetes after approximately 15 years [32]. These results suggest that multiple-risk lifestyle patterns have a synergistic relationship with cardiovascular diseases, such as hypertension and diabetes, compared to low-risk lifestyle patterns [63, 64].

Despite the poorer lifestyle characteristics, the association between hypertension and LCM of low-income group was not significant. Our findings may be partially attributable to the difference in the age distributions of the study participants: a much higher proportion of participants aged 65 years or older in the low-income group than those in the high-income group. Previous studies have indicated that the association between multiple unhealthy lifestyle behaviors and the risk of hypertension is attenuated in older women [65]. *Cohen* et al. [65] stated that the weakened relationship between lifestyle and hypertension with aging can be explained, in part, by physiological differences (e.g., increased arterial stiffness and blunted autonomic nervous system responses to stimuli) among older women. However, considering that mechanisms underlying the differences in susceptibility to hypertension according to income level are complex and unclear [66], further research is needed to fully understand the relationship between hypertension and lifestyle patterns according to income level.

## Practical implications

Although SES plays a significant role in disparities in health and health behaviors from a public health perspective [20], most lifestyle interventions for postmenopausal women do not consider participants’ individual lifestyle patterns and differential pattern characteristics according to income or other SES factors [12, 67]. Lifestyle interventions tailored to the characteristics of the different patterns among low- and high-income postmenopausal women are required.

Since the influence of unmeasured confounding factors has not been determined in this study, practical implications should be discussed cautiously. However, our study highlights the need for a person-centered approach to fully understand the risk of varying patterns of unhealthy lifestyles according to income. Despite the fact that the LP-IFW class does not use substances, health professionals should not consider those in the LP-IFW class in low-income group to be less risky than those in the HD-IF class. The LP-IFW class has multiple risky lifestyle behaviors and is strongly associated with ages 65 years or older and diabetes. These findings suggest that paying more attention to this class is an appropriate strategy to enhance healthy lifestyles. It is important to intensively monitor the lifestyles of elderly and diabetic patients. For both income groups, being employed was significantly related to unhealthy lifestyle patterns but tended to have a greater impact on the patterns in high-income group than in low-income group. Considering these points, health professionals should provide resources or develop interventions to enable postmenopausal working women to engage in healthy lifestyles [61]. In addition, women aged under 65 years in both groups were likely to experience HD-IFS classes; therefore, interventions to address alcohol-related concerns may need to be considered for younger women.

## Strengths and limitations

Our study had several strengths. First, it confirmed the income level-specific lifestyle behavior patterns of postmenopausal women. Second, we performed a secondary analysis of nationally representative data to facilitate generalization to postmenopausal Korean women. Third, we used biometric data for hypertension and diabetes to increase the accuracy of the relationship between the latent class memberships and chronic diseases. *Molenaar* et al. [68] suggested using a self-reported questionnaire with biometric data, because self-reported hypertension and diabetes may underestimate the prevalence.

However, our findings should be interpreted with the following limitations. First, owing to the cross-sectional nature of the data, causal inferences between lifestyle patterns and predictors were not established. Second, it was not possible to use validated lifestyle measures and adjust for potential unmeasured confounding factors because we conducted a secondary data analysis. Third, all lifestyle behaviors were self-reported. Self-reports of substance use may be underreported and those of exercise may be overreported due to social desirability [69].

## Conclusions

This study identified the differential patterns of lifestyle behaviors among low- and high-income postmenopausal women using a person-centered approach. Compared to high-income group, the unhealthy lifestyle patterns of the low- income group appeared to be relatively more complex. Considering the importance of lifestyle behaviors for health and quality of life later in life, it is necessary to provide tailored lifestyle interventions based on the income levels of postmenopausal women. Further research on lifestyle patterns according to SES in postmenopausal women is required. First, although income was used as a proxy variable for SES, future studies using various SES proxy variables are needed to enhance the comprehensive understanding of SES and lifestyle patterns. Second, experimental studies should be conducted to verify the effects of tailored lifestyle interventions that consider the characteristics of lifestyle patterns according to SES in postmenopausal women. Third, the effects of SES-specific lifestyle patterns on diverse health outcomes in postmenopausal women should be confirmed using longitudinal data.

## Data Availability

Data from the KNHANES are publicly available, therefore, any researcher can be obtained after request from the website https://knhanes.kdca.go.kr/knhanes/eng/index.do.

## Abbreviations

SES: Socioeconomic status
LCA: Latent class analysis
LCM: Latent class model
KNHANES: Korea National Health and Nutrition Examination Survey
KDCA: Korea Disease Control and Prevention Agency
PSU: Primary sampling unit
CC: Combustible cigarette
EC: Electronic cigarette
HTP: Heated tobacco product
G^2^: Likelihood-ratio statistics
AIC: Akaike information criterion
BIC: Bayesian information criterion
RH: Relatively healthy
LP-IFW: Lowest physical activity, insufficient fruit intake, and no intention to control weight
HD-IF: High-risk drinking and insufficient fruit intake
LP: Lowest physical activity
HD-IFS: High-risk drinking and insufficient fruit intake and sleep
OR: Odds ratio
